# Foreign Summary

**Published:** 1838-01-03

**Authors:** 


### FOREIGN SUMMARY.

We extract from the "Memoirs of the society of Medical Observation," a notice of the late James Jackson, Jr. M. D., of Boston, from the pen of M. Louis. This tribute to the memory of our lamented young countryman, from such a source, is peculiarly gratifying.

This young physician, son of Professor Jackson, of Boston, fell a victim to a succession of acute attacks, shortly after his return to America, in 1833, leaving his family plunged in the profoundest grief, and sincerely regretted by all who knew him. I have never met with any one, who united greater delicacy of sentiment and loftiness of character, to a sounder mind, a more lively devotion to science, or greater talents for observation. These talents revealed themselves from the period of his entry into this society, of which he was one of the most useful members; and all who knew him, still hold him up to themselves as a model. No one possessed, in a higher degree, the faculty of investing a fact with all that could give it value, or strengthen its claims to reality.

*Chloride of Soda in Typhoid Fever.*—A late number of the Dublin Journal contains some interesting "observations on the use of certain remedies in typhoid fever, and its complications; by Alfred Hudson, M. B. T. C. D. Physician to the Navan Fever Hospital." On the subject of the treatment of Typhoid Fever, he remarks: As a routine practice, I think the solution of chloride of soda is to be preferred to any other, provided that it be given as soon as possible after the type of the fever is known, which, in many cases, means, of course, as soon as the fever has set in. I cannot say that I have seen such good effect from its use in more advanced stages, though I have prescribed it in a large number of such, and still do so; not, however, to the exclusion of any one of our tried and approved remedies. But I have noted forty-seven cases of typhoid fever in which I commenced its use as soon as the patient was admitted, or the type of the fever was evidenced by the appearance of petechiæ, &c., and in every instance with the best effect, this being (in many cases) the only medicine given; the dose was from ten to fifteen drops. In some of these cases, the effect of the chloride was evidenced by the change of colour, and diminution in number of the petechiæ, 'taches rosées,' within twenty-four hours, shewing, I think, that its action is exerted directly upon the blood, and not as a stimulant of the nervous system, as a late writer in the Dublin Journal seems (erroneously surely) to have inferred from Dr. Graves's paper on this subject. For myself, while my limited experience leads me to place the fullest confidence in the chemical effects of this medicine, given early, I have not the least reason to attribute any stimulant powers, nor indeed any good effect whatever, to it in that stage of prostration and adynamia, which Dr. Graves has so graphically described, and in which he considers the chloride a remedy worthy of confidence.

*Death of Dr. Mackintosh of Edinburgh.*—It is with feelings of deep regret that we announce the death of Dr. Mackintosh, by typhus fever, which he was supposed to have caught from a patient. In Dr. Mackintosh, who has been thus prematurely cut off, almost in the prime of life, we have lost one of the most eminent physicians of the present day. He was not only known as one of the first medical authorities in this city, but his reputation may be said to have extended wherever medical science was cultivated.—*Caledonian Mercury.*

### *Analysis of the Memoirs of the Royal Academy of Medicine of Paris.*

From the Edinburgh Med. and Surg. Journal for October, 1837.

*Remarks on superficial cancers. by J. Lisfranc.*—The attentive study of cancerous affections, has convinced the author, that diseases of this kind, do not at once attack all the tissues of a particular part, and that extirpation, when it is had recourse to, may therefore be limited to one portion of an organ, without necessarily involving the whole. Cancer of the stomach he found sometimes limited to the muscular coat, sometimes to the cellular, the pleura, and peritoneum, often oppose a barrier to the extension of cancers of the breast, and umbilicas; and in affections of the penis, the *corpora cavernosa* frequently remained untouched. In applying these principles, the author was enabled, in the case of a diseased penis, by a very careful and slow dissection, to remove the whole of the diseased part, and leave a considerable portion of the organ remaining. The same favorable result attended an operation on the scrotum and on the tongue, and he likewise had it in his power, successfully to remove a cancerous portion from the inferior posterior part of the vagina, of the size of a six franc piece. He proposes a similar operation in carcinomatous affections of the rectum and *os uteri*; and he gives in a subsequent part of the volume, another memoir on the excision of the inferior part of the rectum when cancerous.

In this paper he states, in the first place, his reasons for believing, that an operation of this kind, at the extremity of the rectum, may be practised with safety. The peritoneum terminates at six inches from the anus in women, and at four in men. The vessels at the extremity of the rectum may be opened without much inconvenience, and an oval incision made in the skin which surrounds the anus, which allows a diseased part to be conveniently reached. The cellular tissue which unites the rectum to the vagina in women, and to the urethra in men, allows a ready separation of those organs, with the fore-finger, during an operation, while he considers that there is a sort of second ophineter, above the anus, which may come into use when the other is removed. M. Lisfranc, therefore, felt himself justified in attempting the cure of cancers of the rectum by incision; but he proceeded gradually from the more superficial description, to those which involved a portion of the sphincters, and then to the removal to the extent of even three inches and a half of the inferior orifice of the rectum. He states that of nine cases operated upon, six did well, and three died; but he considers the chances of success to be still greater if taken at an earlier period.

———

*Report on a case of congenital extrophy of the bladder, by M. Velpeau.*—The author and M. Breschet were deputed to report on the case of a child, six days old, born at its full time, which had in the hypogastric region, just below the umbilicus, a red irreducible bleeding fungous tumor, about the size of an egg, which distilled a limpid fluid, from two little papillæ, having all the properties of urine. The tumor was constituted of the posterior part of the bladder, and M. Velpeau gives a detail of many cases of a similar kind which are to be found in authors, and of which he has not met with fewer than one hundred. These cases were long regarded as Hernia of the bladder, but *Tenon* satisfactorily demonstrated the error of this opinion, and showed, that tumors of this kind consist of the posterior part of the bladder, pushed down by the abdominal viscera to supply the loss of substance, which has taken place in the interior part of that organ, and in the parietes of the abdominal cavity below the umbilicus. Defects of this kind are not to be regarded as monstrosities, but as the results of disease; the parietest of the abdomen, in the first instance, and the anterior surface of the bladder, in the next, being destroyed by ulceration; and the posterior surface of the bladder uniting by adhesion with the destroyed edges of the abdominal parietes, and thus presenting externally the spongy mucus surface of the bladder externally.

